# Pharmacogenomics Implementation Training Improves Self-Efficacy and Competency to Drive Adoption in Clinical Practice

**DOI:** 10.3389/fphar.2021.684907

**Published:** 2021-06-28

**Authors:** Fadhli Adesta, Caroline Mahendra, Kathleen Irena Junusmin, Arya Melissa Selva Rajah, Sharon Goh, Levana Sani, Alexandre Chan, Astrid Irwanto

**Affiliations:** ^1^ Nalagenetics Pte Ltd, Singapore, Singapore; ^2^ Department of Clinical Pharmacy Practice, School of Pharmacy and Pharmaceutical Sciences, University of California, Irvine, Irvine, CA, United States; ^3^ Department of Pharmacy, Faculty of Science, National University of Singapore, Singapore, Singapore

**Keywords:** healthcare professionals, education, implementation training, pharmacogenomics, program evaluation

## Abstract

**Background:** Administration of pharmacogenomics (PGx) testing in clinical practice has been suboptimal, presumably due to lack of PGx education. Here, we aim to evaluate the standpoint of PGx testing among a diverse group of healthcare professionals (HCPs) through conducting surveys before and after training.

**Materials and Methods:** Training modules were designed to cover three key learning objectives and deployed in five sections. A pre- and post-training survey questionnaire was used to evaluate participants’ self-assessments on employing PGx in clinical practice.

**Results and Conclusion:** Out of all enrollments, 102 survey responses were collected. Overall, respondents agree on the benefits of PGx testing, but have inadequate self-efficacy and competency in utilizing PGx data. Our results show that a 90 min long training significantly improves these, and could lead to greater anticipation of PGx adoption.

## Introduction

Pharmacogenomics (PGx) focuses on the influence of genetic variations on drug response (Just et al., 2017). PGx is progressing from identifying drug-gene pairs to assimilating into clinical practice (Frick et al., 2016). A recent study conducted in Singapore observed that 30% of adverse drug reactions (ADRs) were caused by at least one drug with a PGx clinical annotation, suggesting the potential to prevent ADR occurrence *via* PGx testing (Chan et al., 2016). Despite PGx testing demonstrating its potential to enhance medication safety and efficacy (Dunnenberger et al., 2015), its utility in clinical practice has been suboptimal (Chan et al., 2017), specifically in Asia (Lee et al., 2017). The lack of PGx education is an often-cited barrier to the widespread implementation of PGx (Mccullough et al., 2011; Kisor et al., 2015; Luzum and Luzum, 2016; Kisor and Farrell 2019).

Although PGx didactic teaching is increasing in undergraduate and postgraduate schools of medicine and pharmacy (Lee et al., 2015; Adams et al., 2016; Frick et al., 2016; Remsberg et al., 2017; Marcinak et al., 2018; Frick et al., 2018; Kisor and Farrell 2019), PGx education is not readily available to practicing clinicians (Kuo et al., 2013; Chan et al., 2017; Kisor and Farrell 2019). Clinicians may not have sufficient training and educational background to offer patient care incorporating PGx and personalized care overall (Kisor et al., 2015; Chan et al., 2017). Consequently, their poor perceived ability to clinically integrate PGx has been widely reported (Mccullough et al., 2011; Stanek et al., 2012; Kuo et al., 2013; Chan et al., 2017; Kisor and Farrell 2019). In particular, a survey on Singaporean clinicians practising in psychiatry observed that only 46.4% of respondents felt competent to order PGx tests (Chan et al., 2017). In this regard, PGx education may help to bridge the knowledge translation gap of PGx use among clinicians (Kuo et al., 2013).

PGx educational courses may be the key to encouraging greater assimilation of PGx into routine practice, having proven to improve attitudes (Luzum and Luzum 2016) and increase the adoption of testing (Owusu-Obeng et al., 2014). A study conducted on physicians observed that a 45 min PGx presentation can improve their attitudes toward PGx testing (Luzum and Luzum 2016). PGx educational courses would have to be constantly updated to ensure sustainable PGx assimilation into routine clinical practice (Dunnenberger et al., 2015; Lee et al., 2017). There have been scarce resources available for doctors to learn pharmacogenomics online. Up to date, there are four major providers (ASHP, ACCP, Mayo Clinic, and NACDS) who have attempted to deliver PGx courses online.

The only accredited online pharmacogenomics certification course is offered by American Society of Health-System Pharmacists (ASHP). However, this course heavily focuses on how to set up a pharmacogenomics practice, i.e., sourcing labs to run samples and obtaining stakeholder approval (Haidar et al., 2015). Our course aims to equip the enrollee with the most relevant clinical knowledge at the least amount of time, without going into the administrative details. Aside from the course provided by ASHP, American College of Clinical Pharmacy (ACCP) provides PGx training course that combines online-delivered materials and workshops to better explain about case studies of PGx in different medical fields. ACCP requires offline in-person workshops, which means that participants are only able to enroll four times within a year (Hicks et al., 2019). This greatly reduces the accessibility of the course, especially for healthcare professionals outside the United States. Mayo Clinic provides general training in clinical pharmacogenomics specifically for physicians. The course is fully online with take home assignments that will be marked manually by the instructor. The 16 h course is disease or condition specific with the bulk of the course the students are taken into different disease types that will benefit from PGx, example one module focusing on pharmacogenomics application in psychiatry while another focuses on its application in cardiology. The course itself is divided into four stages: Principles and concept, general application, case based application, and implementation, with the bulk of the content being in the case based application (Giri et al., 2018). We find that Mayo clinic is the most comprehensive in terms of setting up a good scientific foundation, yet its design is very US-centric, expensive for practitioners in SE Asia, and time consuming. Lastly, there is Test2Learn program that is a joint course jointly developed by NACDS, University of Pittsburgh and with the help of a private genetic testing company 23andMe. The course is heavily focused on immediate application of pharmacogenomics knowledge in relation to the reports provided (Adams et al., 2016), hence there might not be sufficient knowledge on the principles and concepts.

Previous studies have reported an increase in clinical adoption of PGx testing by HCPs to improve prescription outcomes in North America. In the States, Mayo Clinic reports that adoption of pharmacogenomics has increased adoption of its practice in community clinics (Klein et al., 2017; Giri et al., 2018). This finding is supported by another systematic review stating that the main hindrance of the adoption of pharmacogenomics is the rapidly changing landscape of which traditional education may not be able to keep up. However, such reports mainly analyses practices in America and Europe, while studies on Asian practices remain unclear.

The objective of this study was to assess the current level of understanding toward PGx among HCPs practicing in Asia. This was conducted through training materials that were delivered as offline and online courses to healthcare professionals. Here, we aim to evaluate the development and outcomes of a PGx implementation training program. The training program’s objectives were: (1) to understand PGx applications to clinical practice, (2) to be able to engage in patient discussions about PGx testing, and (3) to interpret, evaluate and implement PGx recommendations. Participants’ perceptions of the clinical relevance and utility of PGx, and their self-efficacy and knowledge to integrate PGx into practice were assessed.

## Materials and Methods

### Ethical Approval

This study was approved by the institutional review board (IRB No. 038/KEPK/III/2018 for Indonesia; 2017/007 for Singapore). Written consent was obtained from FGD participants, highlighting voluntary participation.

### Study Recruitment

Participants were HCPs residing in Singapore and Indonesia, including medical doctors, nurses, medical and pharmacy students, and other healthcare related workers that do not fall into a specified position. HCPs were invited to participate in Training Modules (TM) at no cost by announcing the event through email and word-of-mouth invitations and participation was voluntary.

### Offline Training Module 1, TM 1

**FIGURE 1:**
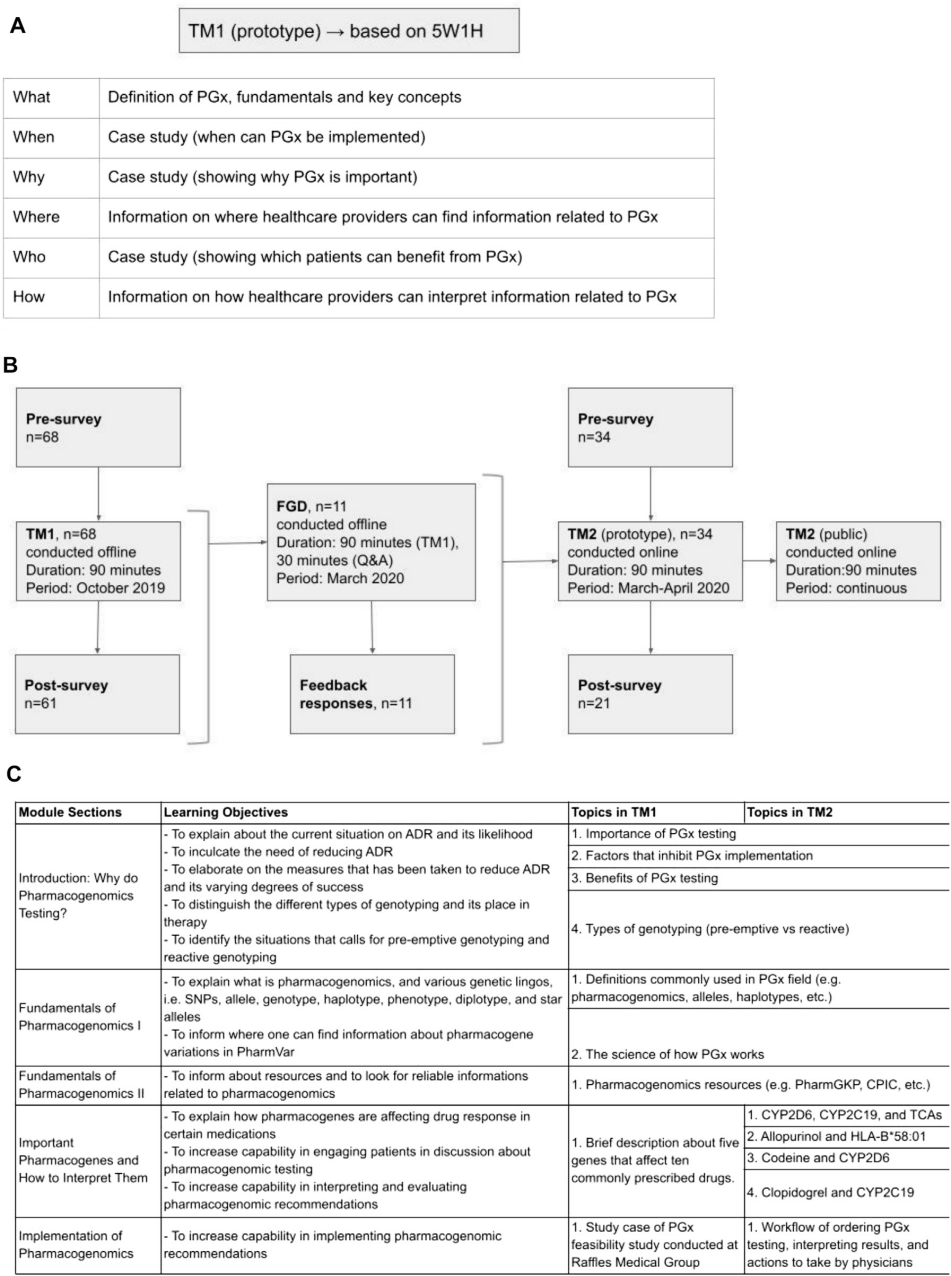
Development of training materials. **(A)** Questions composed of 5W1H (what, when, why, where, who, and how) are used to determine the first training materials and objectives. **(B)** The first training material (TM1) was designed based 5W1H questions as seen on Panel 1A and implemented in an offline training. Focus group discussion (FGD) with the TM1’s training participants was held offline to collect feedback. Feedback from the FGD was used to develop more comprehensive training materials (TM2) and implemented as online training. **(C)** Details of the learning objectives and topics covered in TM1 and TM2.

The “5W1H” approach was adopted for developing the initial training material. Elaborating on the “what, when, why, where, who and how” of PGx systematically introduced fundamental PGx concepts. The training began with “what” PGx is, defining fundamental terminology and key genetic concepts. Information on “where” HCPs may gather relevant PGx information and “how” to maneuver through PGx resources, such as PharmGKB, CPIC, and DPWG, was shared. A patient case was used to consolidate four concepts: “when” PGx testing can be implemented, “why” PGx is important, “how” HCPs can interpret PGx information, and “who” to apply PGx to in clinical settings (Figure 1A).

TM1 was conducted to train physicians practicing in hospitals. TM1 was conducted as an in-person classroom session with 90 min of didactic instructions in Raffles General Hospital, Singapore and Rumah Sakit Cipto Mangunkusumo, Indonesia. Prior to the classroom instruction, the participants were asked to fill in a 10 min survey to determine their baseline perception, efficacy, and competency in pharmacogenomics implementation in clinical practice. Post classroom instructions, 30 min were spared after that to answer questions. A post survey was also conducted after the class over the same set of metrics. Attendance and completion were mandatory except for unforeseen circumstances that require participant to leave the training (Figures 1B,C). Trainee’s participation in both surveys were optional since they were not part of the training content. (Supplementary Appendix S1,S2. TM1 surveys).

### Focus Group Discussion

Participants were invited to voluntarily attend the in-person event by email invitation. The main objective was to collect feedback on TM1, where the first 90 min session was spent on TM1, followed by an open discussion on the training content. The discussion session spanned over 30 min, was audio-recorded, and conducted offline. The FGD was also conducted to evaluate qualitatively participants’ self-perceived competency and efficacy in implementing pharmacogenomics in clinical practice. These metrics were further divided into subcategories of knowledge, comprehension and application questions written in a patient case scenario format. The discussions then further elucidate qualitatively the strength and areas of improvement found in TM1. Questions were tailored to explore the participants’ views on the fundamentals of PGx, PGx applications and course delivery, and were structured in an open-ended format to facilitate rich discussion. The sequence of questions mirrored the sequence of TM1 content. Additionally, the free-flowing nature of the discussion allowed participants to voice suggestions on supplementary aspects of the training. Feedback collected during this FGD was used to improve TM1. To develop TM2, responses from this offline FGD were collected and considered as requests to incorporate into TM2 training structure (Figure 1B).

### Online Training Module 2, Training Modules 2

Based on responses collected during FGD, TM2 was developed to have more in-depth materials, specifically for examples of drug-gene interactions commonly found in clinical settings. More details on the science of PGx and drug-gene interactions were also included in TM2. (Supplementary Appendix S5. TM2 course content).

TM2 was delivered as a 90 min-long online course *via* a private e-learning platform. Data collection was done on willing participants who enrolled within a period of one month since the launch of this course (March 2020–April 2020). For consistency in evaluating the effectiveness of the training material, a similar 5-point Likert-type scale was also employed pre- and post-training on a set of questions pertaining to perception and utility of PGx, self-efficacy on the implementation of PGx in clinical setting and proficiency of applying PGx into practice (Figures 1B,C). Completion of training was compulsory to earn training certificate, however surveys were optional. (Supplementary Appendix S3, S4. TM2 surveys).

### Data Collection

Data collected was used to characterize the participants. To determine whether training could change clinical practice behavior, the post-training survey asked about experience with and anticipation of using PGx tests. An open-ended section was incorporated in order to solicit feedback on the training course content and delivery to validate TM2 and facilitate future PGx educational programs.

#### Surveys incorporated the following aspects:

##### Perceptions

To assess training objective (1), we evaluated for a perception change in clinical relevance (P1) and utility (P2) of PGx. Questions asked in this section are related to how and in what way can PGx be useful in the subjects’ clinical practice.

##### Self-Efficacy

Evaluation of how to utilize PGx data in making drug therapy decisions (SE1) and how to engage in patient discussion about PGx (SE2) were necessary to assess training objectives (2) and (3). Questions asked in this section are related to how competent the subjects feel about implementing PGx practice.

##### Knowledge

Knowledge, comprehension and application questions regarding clinical PGx recommendations were crafted as a patient case scenario to evaluate for training objective (3). Knowledge assessments were adapted from ASHP’s pharmacogenomics professional certification course. Questions assessing the knowledge taught in our self-developed PGx course were designed by licensed pharmacists who had undergone this ASHP’s certification course. This section also included a case study example. The aforementioned concepts were adopted from the first three levels of Bloom’s taxonomy, i.e., knowledge (remembering), comprehension (understanding), and application (applying).

### Data Analysis

Ordinal data related to participants’ perceptions and self-efficacy were summarized using median and interquartile range (IQR). Items assessed on a five-point Likert scale were collapsed and presented as the percentage of agree, disagree and neutral responses. Distribution of responses between the pre- and post-training surveys were compared using the Wilcoxon’s rank sum test. Knowledge questions were scored as correct or incorrect, with missing answers scored as incorrect. The percentage of correct responses overall and for each question on the pre- and post-training surveys were compared using chi-square test. Statistical analyses were conducted using R Version 3.5.2, with *p* < 0.05 considered as statistically significant.

## Results

### Structure of Training Module 1

The content of training materials focused on PGx applications. Training outcomes were based on the competency inventory curated by the Pharmacogenetics/Pharmacogenomics Special Interest Group of the American Association of Colleges of Pharmacy (Roederer et al., 2017). The training objectives were: 1) to understand PGx applications to clinical practice, 2) to be able to engage in patient discussions about PGx testing, and 3) to interpret, evaluate and implement PGx recommendations (Figure 1C). Out of all the participants who attended TM1, 68 and 61 survey responses were collected pre- and post-training (Figure 1B).

### Demographic of Respondents in Training Module 1

TM1 respondents consisted of 93.4% physicians, of which 68.9% practiced in Family Medicine and 18.9% did not state their specialty, while the remaining 6.6% practiced in surgery, cardiology, and other specialties (Table 1). More than half, 55.9%, had more than 5 years of practice experience. Prior experience in PGx education was lacking across the respondents, where only 27.9% responded having received PGx education before this training. This includes self-learning from independent resources (internet, colleagues, journals, drug labels or package inserts), attending a lecture or seminar, and/or enrolling in university curriculum. There was lack of enrollment from other HCPs like nurses and pharmacists during this training module since the target audience were practicing doctors at a hospital.

**TABLE 1:** Participant characteristics in offline TM1 training.

Characteristics	Offline; TM1			
	Pre, *n* = 68		Post, *n* = 61	
	n	%	n	%
Age (mean and range)	42.1	(24–73)	43.1	(24–73)
Gender				
Male	33	48.5	32	52.5
Female	28	41.2	21	34.4
Position				
Doctor	63	92.6	57	93.4
Pharmacist	4	5.9	3	4.9
Nurse	0	0.0	0	0.0
Medical student	0	0.0	0	0
Pharmacy student	0	0.0	0	0
Others	1	1.5	1	1.6
Specialty				
Family medicine	44	64.7	42	68.9
Surgery	1	1.5	2	3.3
Emergency medicine	1	1.5	0	0.0
Others	8	11.9	3	4.8
Not applicable	1	1.5	1	1.6
No response	13	18.9	13	21.4
Practice experience				
1–5 years	18	26.5	15	24.6
6–10 years	11	16.2	10	16.4
11–20 years	12	17.6	10	16.4
21–30 years	11	16.2	9	14.8
31–40 years	3	4.4	4	6.6
41–50 years	1	1.5	1	1.6
No response	12	17.6	12	19.6
Previous experience with PGx education	19	27.9		

### Pre- and Post- Perception, Self-Efficacy and Knowledge in Training Module 1

#### Relevance and Utility of Pharmacogenomics Testing in Clinical Practice

**FIGURE 2:**
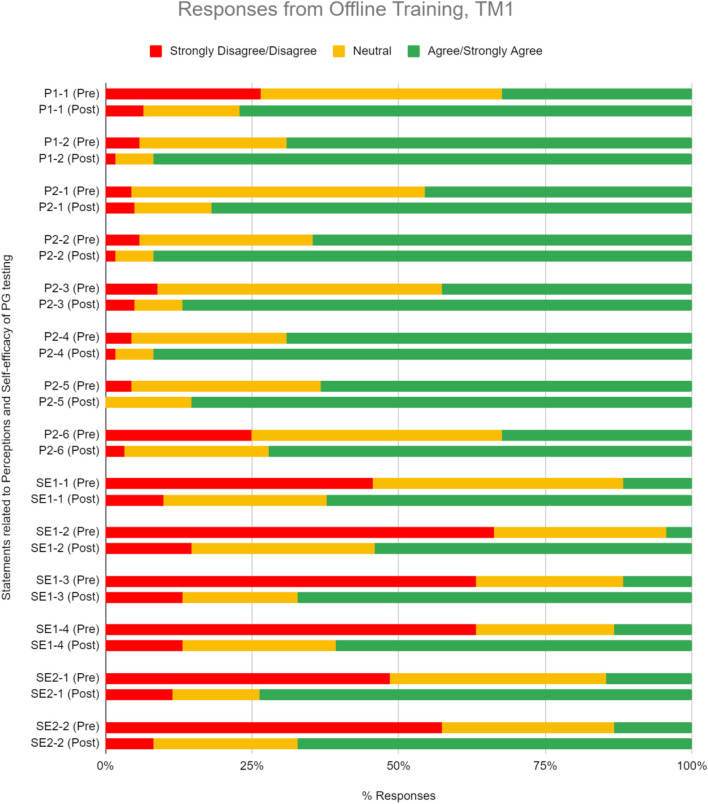
Percent of respondents relative to 5-point Likert-type scale labels pre- and post-PGx training conducted offline for TM1 in perception (P) and self efficacy (SE) sections. TM1 pre-survey, *n* = 68; TM1 post-survey, *n* = 61.

We inquire on a set of perception questions pre- and post-training, relative to 5-point Likert-type scale (Table 2). Prior to training, 52.4% TM1 participants generally agree or strongly agree with the clinical relevance and utility of PGx testing, indicating favorable perceptions toward PGx (Figure 2). This number increased even more after training to 84.8% in TM1. Overall, participants’ median scoring in perceptions for TM1 improved from 3 to 4 (Table 2; *p* < 0.05), suggesting statistically significant positive perception change upon completion ofTM1 training. Notably, 77% from TM1 respondents reported greater anticipation of using PGx tests after attending the training.

**TABLE 2:** Pre- and post-offline TM1 training results related to perceptions and self-efficacy of addressing PGx testing.

Survey items	Offline; TM1				
	Pre, *n* =68		Post, *n* =61		p-value^c^
	Median score^a^	IQR^b^	Median score^a^	IQR^b^	
Perceptions (P1): Relevance of PGx to clinical practice					
P1-1 PGx is relevant to my clinical practice/I am keen in adopting PGx into my clinical practice	3	2	4	0	< 0.05
P1-2 I believe that a patient’s genetic profile may influence his/her response to drug therapy	4	1	4	1	< 0.05
Perceptions (P2): Clinical utility of PGx					
P2-1 In general, the benefits of PGx testing outweigh the risks	3	1	4	1	< 0.05
P2-.. PGx testing is useful for...					
P2-2 ...identifying suitable medications for treatment	4	1	4	1	< 0.05
P2-3 ...guiding dosing of medications	3	1	4	1	< 0.05
P2-4 ...reducing adverse drug reactions	4	1	4	1	< 0.05
P2-5 ...improving treatment efficacy	4	1	4	1	< 0.05
P2-6 ...reducing treatment costs	3	1	4	2	< 0.05
Self-efficacy (SE1): Perceived belief in ability to use PGx information to guide drug therapy decisions					
SE1- I feel competent in					
SE1-1 identifying clinical situations and/or patients in which PGx testing is indicated	3	1	4	1	< 0.05
SE1-2 interpreting PGx test results	2	1	4	1	< 0.05
SE1-3 making treatment recommendations based on PGx test results	2	1	4	1	< 0.05
SE1-4 I can identify good PGx resources (e.g., guidelines) for use clinically	2	1	4	1	< 0.05
Self-efficacy (SE2): Perceived belief in ability to engage in patient discussion about PGx testing					
SE2-1 I feel competent in explaining the rationale of PGx testing to patients	3	1	4	1	< 0.05
SE2-2 I feel competent in discussing the risks and benefits of PGx testing with patients	2	1	4	1	< 0.05

a Score is ranged using five-point Likert scale from (1) strongly disagree to (5) strongly agree.

b IQR is calculated as the difference in scores falling in the first and third quartile.

c Wilcoxon test was used to analyze changes in pre- and post-training responses. Significant p-values (<0.05) are bolded.

#### Perceived Ability in the Implementation of Pharmacogenomics in Clinical Setting

We inquire on a set of self-efficacy questions on the perceived belief in ability to use PGx information to guide drug therapy decisions and engagement in patient discussion about PGx testing (Table 2). Pre-training results demonstrate that respondents begin with inadequate self-efficacy in using PGx data to guide medication therapy and engage with patients. Upon completion, training increased their perceived ability in implementing PGx by 51.5% in TM1 (Figure 2). Participants median scoring in the self-efficacy section improved significantly from 2 to 4 in TM1 (Table 2; *p* < 0.05).

#### Knowledge and Proficiency in Applying Pharmacogenomics to Practice

**FIGURE 3:**
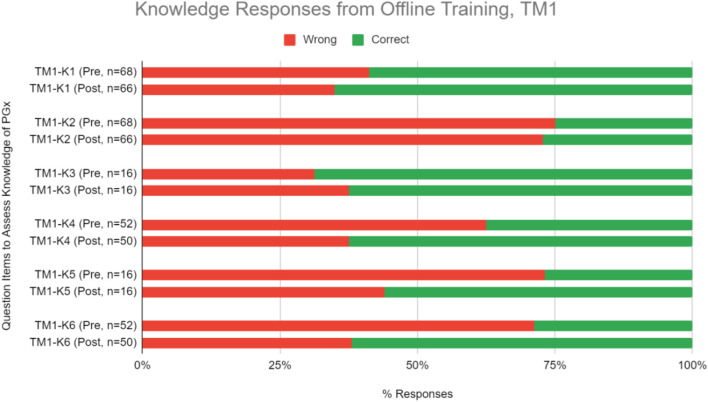
Percent of correct or wrong responses to knowledge questions obtained during pre-and post-PGx offline training TM1.

Questions to evaluate knowledge gain were categorized under two sets–theoretical PGx and practical clinical implementation of PGx. Respondents were quizzed pre- and post-training, and their performance was assessed to evaluate improvements (Table 3). On average, respondents significantly improved their correct response rate for proficiency questions by 15.1% in TM1 (Figure 3, Table 3; Chi-square test, *p* < 0.05). Training has statistically significant improvements in scores for one knowledge level question under theoretical PGx category. On the practical implementation of PGx, a significant improvement is seen for an application level in TM1 (Figure 3, Table 3).

**TABLE 3:** Correct responses to questions about knowledge on PGx comparing pre- and post- offline training for TM1.

Survey questions	Correct answer	Correct rResponses				
		Offline; TM1				
		Pre		Post		p-value
		n	%	n	%	
Knowledge (K1): Knowledge on theoretical PGx						
K1-1. What may be the consequence of a PGx polymorphism? (comprehension level)	An individual has a higher risk for toxicity when using prescription drugs.	40	59	43	65	0.4507^a^
K1-2. What does a poor metabolizer phenotype indicate? (knowledge level)	Decreased enzyme activity.	17	25	18	27	0.7646^a^
K1-3. A patient with CYP2D6 activity score of 1.5 has which CYP2D6 phenotype? (knowledge level)	Normal metabolizer.	6	38	10	63	0.1573^a^
K1-4. Which of the following is not correct about pre-emptive and reactive genotyping? (knowledge level)	Reactive genotyping has been shown to be more cost-effective than pre-emptive genotyping.	14	27	28	56	< 0.05^a^
K1-5. What does an ultra-rapid metabolizer phenotype for CYP2C19 indicate? (knowledge level)	Increased enzyme activity					
Knowledge (K2): Practical clinical implementation of PGx						
K2-1. There is a high chance that Ms Lee will develop Stevens Johnsons Syndrome? (comprehension level)	FALSE					
K2-2. What is Ms Lee’s CYP2D6 enzyme activity score? (comprehension level)	2					
K2-3. Which of the following would be appropriate regarding Ms Lee's amitriptyline therapy according to CPIC guidelines? (Aapplication level)	Consider alternative drug not metabolized by CYP2C19					
K2-4. A woman is diagnosed with breast cancer and, as part of her oncology regimen, she is treated with tamoxifen. She did not have genetic testing performed before initiating treatment. What PGx reason would cause the treating physician to decide to change the drug? (application level)	CYP2D6 poor metabolizer resulting in lack of drug.	11	69	10	63	0.7097^a^
K2-5. Which of the following would be appropriate regarding clopidogrel therapy in patients who are CYP2C19 poor metabolizers? (application level)	Consider alternative antiplatelet therapy if no contraindications.	15	29	31	62	< 0.05^a^

a Chi-square test was used to compare the percentage of correct responses between pre- and post- training surveys. Significant p-values (<0.05) are bolded.

b Fisher’s test was used if criteria for Chi-square (expected value size > 5) is not met.

### Focus Group Discussion (FGD) Structure and Analysis of User Responses

**FIGURE 4:**
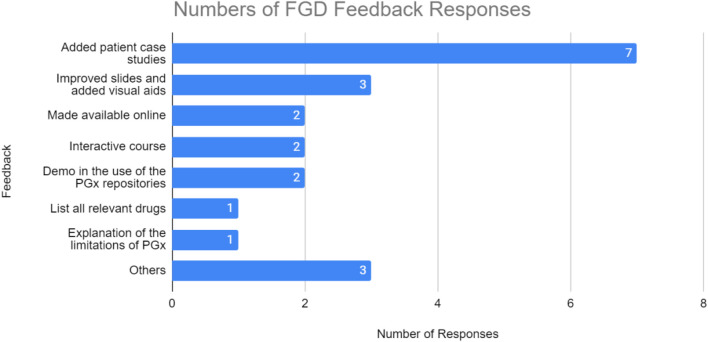
Number of responses from focus group discussion feedback. TM1 participants highly suggested more case studies to be added on the training material.

Most of the FGD responses revolve around the request to add patient case studies (seven times mentioned). Two of the responses also requested that the test be available online, so that they can replay and reassess the video when required. Additionally, the focus group mentioned the need for more visual aids (three mentions), a demo in the use of the PGx resources (two mentions), including the list of relevant drugs as well as to make the course more interactive (two mentions). Others refer to requests that are not relevant for improvement (Figure 4).

### Development and Structure of TM2

To facilitate improvements that capture the most common requests identified during the FGD discussion, TM2 was delivered as an online course. This also allows for more flexibility in terms of time and place needed to complete the whole material, as well as scalability (Figures 1B,C).

### Demographics of Respondents in TM2

TM2 attracted a more diverse demographic of healthcare professionals, likely due to its online nature (Table 4). The resulting participant pool consisted of physicians (61.8%), pharmacy students (20.6%), medical students (8.8%), practicing nurses (5.9%), and pharmacists (2.9%). Only two medical specialties were represented (Family Medicine and Emergency Medicine) and almost all participants have a practicing experience of less than five years. Similar to TM1, there was a lack of previous experience with pharmacogenomics.

**TABLE 4:** Participant characteristics in online TM2 training.

Characteristics	Online; TM2			
	Pre, *n* = 34		Post, *n* = 21	
	n	%	n	%
Age (mean and range)	30.41	(23–50)	29.69	(23–46)
Gender				
Male	19	55.9	10	47.6
Female	15	44.1	11	52.4
Position				
Doctor	21	61.8	8	38.1
Pharmacist	1	2.9	1	4.8
Nurse	2	5.9	2	9.5
Medical student	3	8.8	3	14.3
Pharmacy student	7	20.6	7	33.3
Others	0	0.0	0	0.0
Specialty				
Family medicine	21	61.8	8	38.1
Emergency medicine	2	5.9	2	9.5
Others (surgery, cardiology, etc.)	0	0.0	0	0.0
Not applicable	11	32.4	11	52.4
Practice experience				
1–5 years	10	29.4	6	28.6
6–10 years	4	11.8	2	9.5
11–20 years	7	20.6	3	14.3
21–30 years	3	8.8	0	0.0
>30 years	0	0.0	0	0.0
No response	10	29.4	10	47.7
Previous experience with PGx education	11	32.4		

### Pre- and Post- Perception, Self-Efficacy and Knowledge in Training Module 2

#### Relevance and Utility of Pharmacogenomics Testing in Clinical Practice

**FIGURE 5:**
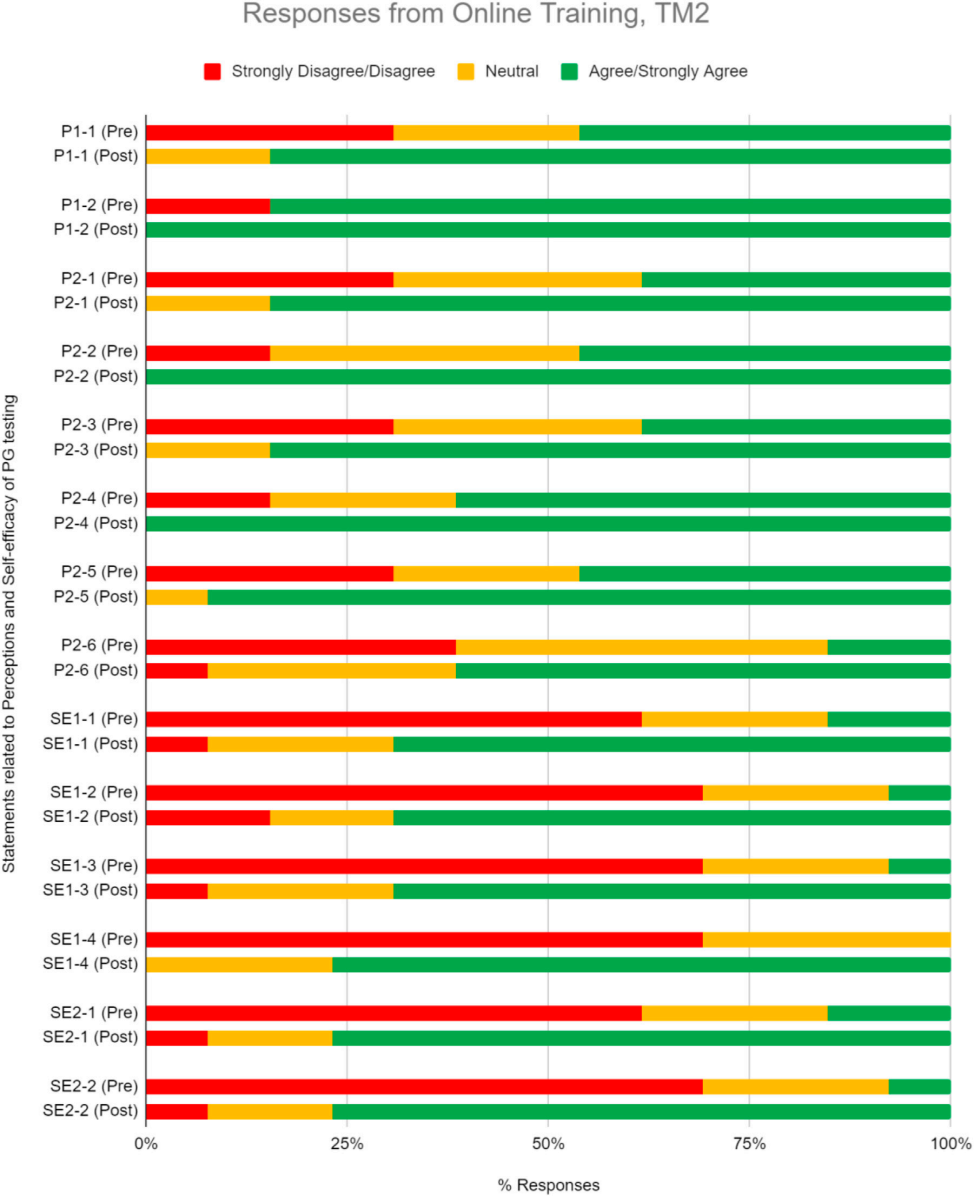
Percent of respondents relative to 5-point Likert-type scale labels pre- and post-PGx training conducted online for TM2 in perception (P) and self efficacy (SE) sections. Three questions in P section (P1-2, P2-2, and P2-4) received 100% of respondents answering agree/strongly agree in the post-training questionnaire. TM2 pre-training survey knowledge evaluation, *n* = 34; TM2 post-training survey knowledge evaluation, *n* = 21.

As was seen during TM1, most TM2 respondents agree or strongly agree on the clinical relevance and utility of PGx testing, where the numbers were 62.5% before TM2 training and 88.1% after (Figure 5, Table 5). The median scoring in perceptions remained high at 4 (Table 5; *p* < 0.05). 85.7% of respondents were convinced of the utility of PGx tests after attending the training.

**TABLE 5:** Pre- and post-online TM2 training results related to perceptions and self-efficacy of addressing PGx testing.

Survey items	Online; TM2					
	Pre, *n* = 34		Post, *n* = 21			p-value^c^
	Median score^a^	IQR^b^	Median score^a^	IQR^a^		
Perceptions (P1): Relevance of PGx to clinical practice						
P1-1 PGx is relevant to my clinical practice/I am keen in adopting PGx into my clinical practice	4	1	4		1	< 0.05
P1-2 I believe that a patient’s genetic profile may influence his/her response to drug therapy	4	0	4		1	< 0.05
Perceptions (P2): Clinical utility of PGx						
P2-1 in general, the benefits of PGx testing outweigh the risks	4	1	4		0	< 0.05
P2-.. PGx testing is useful for…						
P2-2 ...identifying suitable medications for treatment	4	1	4		1	< 0.05
P2-3 ...guiding dosing of medications	4	1	4		1	< 0.05
P2-4 ...reducing adverse drug reactions	4	0	5		1	< 0.05
P2-5 ...improving treatment efficacy	4	1	5		1	< 0.05
P2-6 ...reducing treatment costs	3	1	4		1	0.1092
Self-efficacy (SE1): Perceived belief in ability to use PGx information to guide drug therapy decisions						
SE1-.. I feel competent in...						
SE1-1 ...identifying clinical situations and/or patients in which PGx testing is indicated	3	2	4		1	< 0.05
SE1-2 ...interpreting PGx test results	3	1	4		1	< 0.05
SE1-3 ...making treatment recommendations based on PGx test results	3	1	4		1	< 0.05
SE1-4 I can identify good PGx resources (e.g., guidelines) for use clinically	3	2	4		1	< 0.05
Self-efficacy (SE2): Perceived belief in ability to engage in patient discussion about PGx testing						
SE2-1 I feel competent in explaining the rationale of PGx testing to patients	3	2	4		0	< 0.05
SE2-2 I feel competent in discussing the risks and benefits of PGx testing with patients	3	2	4		1	< 0.05

a Score is ranged using five-point Likert scale from (1) strongly disagree to (5) strongly agree.

b IQR is calculated as the difference in scores falling in the firstAnd thirdquartile.

c Wilcoxon test was used to analyze changes in pre- and post-training responses. Significant p-values (<0.05) are bolded.

#### Perceived Ability in the Implementation of Pharmacogenomics in Clinical Setting

TM2 training increased respondents’ perceived ability in implementing PGx by 44.6%, where the median scoring improved statistically from 3 to 4 (Figure 5, Table 5; *p* < 0.05).

#### Knowledge and Proficiency in Applying Pharmacogenomics to Practice

**FIGURE 6:**
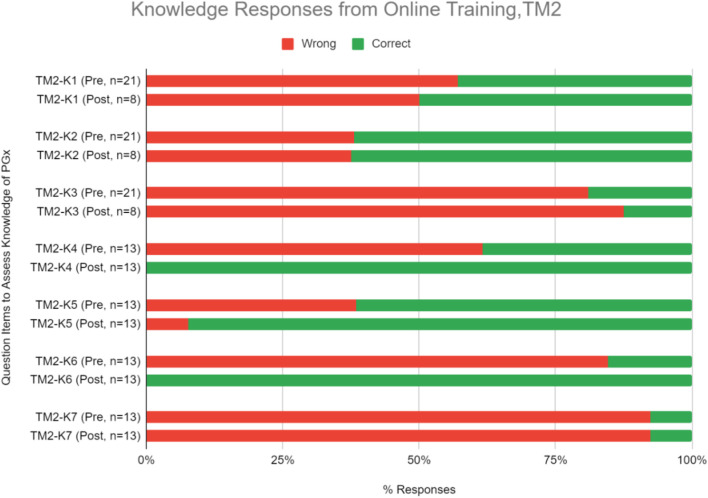
Percent of correct or wrong responses to knowledge questions obtained during pre-and post-PGx online training TM2. Two questions in the knowledge section (TM2-K4 and TM2-K6) received 100% of respondents answering correctly in the post-training assessment.

Knowledge and proficiency in applying pharmacogenomics to practice. This online course also significantly improved respondents’ correct response rate for proficiency questions by an average of 28.0% (Figure 6, Table 6; *p* < 0.05). On the theory of PGx, improvement in scores were seen in at least one knowledge level question while on the practical implementation of PGx, improvement was seen in questions under the comprehension level (Figure 6, Table 6).

**TABLE 6:** Correct responses to questions about knowledge on PGx comparing pre- and post-online training for TM2.

Survey questions	Correct answer	Correct responses				
		Online; TM2				
		Pre		Post		p-value
		n	%	n	%	
Knowledge (K1): Knowledge on theoretical PGx						
K1-1. What may be the consequence of a PGx polymorphism? (Comprehension level)	An individual has a higher risk for toxicity when using prescription drugs	9	43	4	50	1^b^
K1-2. What does a poor metabolizer phenotype indicate? (knowledge level)	Decreased enzyme activity	13	62	5	63	1^b^
K1-3. A patient with CYP2D6 activity score of 1.5 has which CYP2D6 phenotype? (knowledge level)	Normal metabolizer					
K1-4. Which of the following is not correct about pre-emptive and reactive genotyping? (knowledge level)	Reactive genotyping has been shown to be more cost-effective than pre-emptive genotyping	4	19	1	13	1^b^
K1-5. What does an ultra-rapid metabolizer phenotype for CYP2C19 indicate? (knowledge level)	Increased enzyme activity	5	38	13	100	<0.05^b^
Knowledge (K2): Practical clinical implementation of PGx						
K2-1. There is a high chance that Ms Lee will develop Stevens Johnsons Syndrome? (Comprehension level)	FALSE	8	62	12	92	0.1602^b^
K2-2. What is Ms Lee's CYP2D6 enzyme activity score? (Comprehension level)	2	2	15	13	100	<0.05^a^
K2-3. Which of the following would be appropriate regarding Ms Lee's amitriptyline therapy according to CPIC guidelines? (Application level)	Consider alternative drug not metabolized by CYP2C19	1	8	1	8	1^b^
K2-4. A woman is diagnosed with breast cancer and, as part of her oncology regimen, she is treated with tamoxifen. She did not have genetic testing performed before initiating treatment. What PGx reason would cause the treating physician to decide to change the drug? (Application level)	CYP2D6 poor metabolizer resulting in lack of drug					
K2-5. Which of the following would be appropriate regarding clopidogrel therapy in patients who are CYP2C19 poor metabolizers? (Application level)	Consider alternative antiplatelet therapy if no contraindications					

a Chi-square test was used to compare the percentage of correct responses between pre- and post-training surveys. Significant p-values (<0.05) are bolded.

b Fisher’s test was used if criteria for Chi-square (expected value size >5) is not met.

## Discussion

This study evaluated the outcomes of PGx implementation training which was piloted at Continuing Education (CE) seminars and further developed into an online training module. The aim of the training was to educate respondents about the fundamental PGx theoretical concepts and clinical applications. Here, we found that respondents displayed positive perceptions of the clinical relevance and utility of PGx. We also demonstrated that PGx implementation training conducted as a case-based presentation can improve self-efficacy to clinically apply PGx information and to engage in PGx discussions.

### Comparison of Training Module 1 and Training Module 2

The demographics of participants in TM1 showed more numbers of physicians taking the course compared to other healthcare provider positions. This differs from the demographics of TM2 participants which consists of mostly students. This may be due to TM2 being conducted as an online course, thus encouraging students to participate as an additional skill set. TM1 and TM2 also have different numbers of participants. TM1 had more respondents likely because participants were mandated to attend the training in person as a part of their routine Continuing Medical Education (CME) program. On the other hand, TM2 was delivered as an online course which has higher chances of participants not completing the training due to its highly flexible delivery method where participants can decide to pause or stop the training anytime. Less incentive or motivation to participate in an online course aside from personal willingness may also contribute as a factor of TM2 having a lower number of participants.

TM1 seemed to be more attractive to HCPs who were active practitioners in clinical settings. As most active practitioners spend most of their working time in the healthcare facilities, joining a scheduled on-site training program would be more convenient for this group of participants. The disadvantage of this delivery method was that the training program will be limited to participants who are available at the scheduled time, while other practitioners that are unavailable will not be able to get the same learning experience. Conversely, TM2 provided flexibility in participation, thus allowing participants to get a similar learning experience while choosing their own available time and at the convenience of their homes or chosen venue. Online delivery method also allowed participants to replay certain parts that require a deeper level of comprehension. This flexibility increased the materials delivery, thus increasing the efficacy of PGx testing implementation.

### Changes Between Pre- and Post-training for Perception, Self-Efficacy, and Knowledge in Training Module 1 and Training Module 2

**FIGURE 7:**
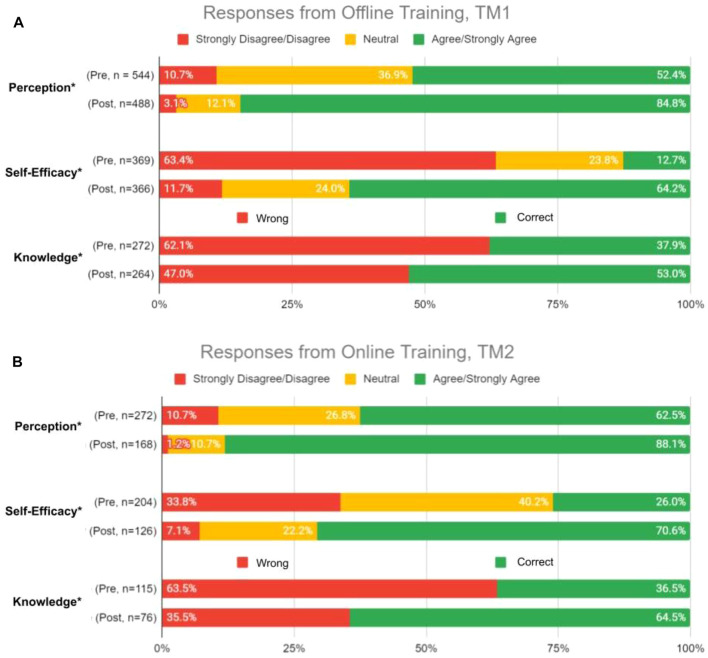
Overall percent of respondents relative to 5-point Likert-type scale labels pre- and post-PGx training conducted **(A)** Offline, TM1; and **(B)** Online, TM2. Survey domains included Perceptions of the relevance and clinical utility of PGx, Self-Efficacy through perceived ability to use PGx information in guiding medical decisions and engage in patient discussions about PGx testing, and Knowledge questions on the PGx-based case studies. Sample size, n, displayed the total number of responses received per domain during surveys pre- and post-training. *Statistically significant p-values (*p* < 0.05) were observed in changes from pre- and post-training responses.

Changes in perception, self-efficacy, and knowledge between pre- and post-test in TM1 showed 32.4, 52.7, and 14.9% increase in agree/strongly agree and correct responses, respectively (Figure 7A). Higher numbers were obtained by TM2, with 41.3, 64.1, and 44.3% increase of agree/strongly agree and correct responses between pre- and post-test in perception, self-efficacy, and knowledge, respectively (Figure 7B). These results demonstrated that TM2 has better potential in improving the participants’ perception, self-efficacy, and knowledge of PGx, making online delivery methods to be more effective in increasing HCPs confidence in implementing PGx.

Some statements and questions of perception and knowledge in TM2 also had 100% rate of agree/strongly agree and correct responses respectively in the post-test. Three perception statements that received 100% agree/strongly agree responses were related to the belief of genetic profile’s influence in drug therapy response and the usefulness of PGx in identifying suitable medications and reducing adverse drug reactions (Figure 5, Table 5). This supported the idea that online delivery methods may be more effective in increasing HCP’s perception of PGx usability. Half of the questions in the TM2 post-test knowledge section which were related to case studies of PGx implementation received 100% of correct answers (Figure 6, Table 6). This indicated that online delivery methods were more suitable in increasing HCP’s skills and knowledge related to implementation of PGx in real clinical settings.

There were improvements in knowledge questions when comparing pre- and post-training responses. Post-offline training, marginal improvements in participants’ knowledge were similarly observed after a PGx educational program for pharmacists (Figure 7A; Formea et al., 2013). On the other hand, post-online training significantly improved the correct response rate (Figure 7B). Limited knowledge retention could be the culprit in subpar improvements for the offline training due to the complexity of PGx or the transfer of overwhelming information over a short period (Formea et al., 2013; Adams et al., 2016). Revisions made to TM2 addressed these pitfalls. Online training materials promote active learning (Gleason et al., 2011) where participants can playback content to enhance knowledge retention. Moreover, complex PGx concepts were more thoroughly explained and quizzes helped to reinforce internalization of content. This was supported by our findings that all participants agreed that the quizzes helped in understanding PGx concepts. Additionally, the online training results reflected a more holistic improvement as the questions were formatted as a case scenario. This has been shown to be more effective for enhancing learning (Gleason et al., 2011).

### Comparison of Our Online Training Material With Other Available Online PGx Courses

TM2 online course was able to fill in the gaps identified in other available courses like ASHP, ACCP, Mayo Clinic, and NACDS. These mentioned online courses require at least 16 h to complete, while our online training material only lasted for approximately 1.5 h to complete. Aside from the training duration comparison, the difference of the courses’ costs were also significant. While other courses have the price ranging from USD $400-$1,099; our training only cost USD $14.85. Lastly, the other mentioned online courses only have pharmacists or physicians as the sole target audience, while our online training material attempted to give PGx-related clinical knowledge that were relevant to all healthcare professionals including physicians, pharmacists, nurses, and students.

## Limitations

Our study had several possible limitations. In both training modules, the pre- and post-training survey responses were unlinked. Consequently, we could not analyze changes to individual responses and could only report aggregate data. Our study population was non-randomized and formed a convenient sample, which may imply selection bias for only respondents with PGx interests. While TM1 offline training involved mainly physicians, with limited participation from four pharmacists, TM2 online training had a lower response rate. This could limit the generalizability of our results to the broader population of clinicians. Results from pre- and post-surveys between TM1 and TM2 could also have been impacted by the mode of delivery (offline vs. online). Finally, actual implementation of testing and the long-term effects of training were not evaluated. This was because the study was intended to provide baseline and initial assessments of the outcomes of PGx implementation training for clinicians. Therefore, we suggest conducting future studies to follow HCPs over a prolonged period to evaluate the effectiveness of regular PGx educational programs and actual clinical update of PGx integration.

## Conclusion

Overall, respondents have favorable perceptions toward PGx testing, but lack self-efficacy and competency in PGx data utilization. Training has been proven to significantly improve self-efficacy and competency. Furthermore, surveys on perception questions revealed that training could lead to greater anticipation of PGx adoption in clinical practice as seen in the increase of agreeable responses to PGx utility. Online training delivery mode is evidently preferable for further improvements. With its flexibility and scalability, it can be expanded as continuous education over a prolonged period to evaluate the effectiveness of PGx education and integration into clinical practice.

## Future Work

Online training delivery mode is evidently preferable for further improvements. With its ability to be flexible and scalable, it can be expanded as continuous education over a prolonged period to evaluate the effectiveness of PGx education and integration into clinical practice.

## Data Availability

The original contributions presented in the study are included in the article/Supplementary Material, further inquiries can be directed to the corresponding authors.
