# Supplementary material for: Pharmacogenomics Implementation Training Improves Self-Efficacy and Competency to Drive Adoption in Clinical Practice
Source: Front Pharmacol. 2021 Jun 28;12:684907. doi: 10.3389/fphar.2021.684907 (PMC8273230; doi:10.3389/fphar.2021.684907)
Supplement: Supplementary file 1 [file DataSheet1.docx]

**SUPPLEMENTARY MATERIAL**

**Appendix 1: Pre-training survey for TM1**

**Survey 1:** Pre-training pharmacogenomics survey

**Introduction**

Pharmacogenomics is the study of how genes affect an individual’s response to drugs. This survey aims to examine clinicians’ knowledge and perceptions of the clinical use of pharmacogenomics testing. This survey should take approximately 5 minutes to complete; thank you for your time.

Please rate the following (circle your answer):

**Section 1: Perceptions**

1. Pharmacogenomics is useful to my current practice.

| Strongly Disagree                                                                        Strongly Agree | | | | |
| --- | --- | --- | --- | --- |
| 1 | 2 | 3 | 4 | 5 |

1. I believe that a patient’s genetic profile may influence his/her response to drug therapy.

| Strongly Disagree                                                                        Strongly Agree | | | | |
| --- | --- | --- | --- | --- |
| 1 | 2 | 3 | 4 | 5 |

1. In general, the benefits of pharmacogenomics testing outweigh the risks.

| Strongly Disagree                                                                        Strongly Agree | | | | |
| --- | --- | --- | --- | --- |
| 1 | 2 | 3 | 4 | 5 |

1. Is pharmacogenomics testing useful for the following?

|  | Not useful at all                                 Extremely useful | | | | |
| --- | --- | --- | --- | --- | --- |
| (a) Identifying suitable medications for treatment | 1 | 2 | 3 | 4 | 5 |
| (b) Guide dosing of medications | 1 | 2 | 3 | 4 | 5 |
| (c) Reducing adverse drug reactions | 1 | 2 | 3 | 4 | 5 |
| (d) Improving treatment efficacy | 1 | 2 | 3 | 4 | 5 |
| (e) Reducing treatment costs | 1 | 2 | 3 | 4 | 5 |

**Section 2: Ability**

1. Please rank your perceived ability:
2. I feel competent in identifying clinical situations and/or patients in which pharmacogenomics testing is indicated.

| Strongly Disagree                                                                        Strongly Agree | | | | |
| --- | --- | --- | --- | --- |
| 1 | 2 | 3 | 4 | 5 |

1. I feel competent in interpreting results of pharmacogenomics tests.

| Strongly Disagree                                                                        Strongly Agree | | | | |
| --- | --- | --- | --- | --- |
| 1 | 2 | 3 | 4 | 5 |

1. I feel competent in making treatment recommendations based on results.

| Strongly Disagree                                                                        Strongly Agree | | | | |
| --- | --- | --- | --- | --- |
| 1 | 2 | 3 | 4 | 5 |

1. I can identify good pharmacogenomics resources (e.g. guidelines) for use clinically.

| Strongly Disagree                                                                        Strongly Agree | | | | |
| --- | --- | --- | --- | --- |
| 1 | 2 | 3 | 4 | 5 |

1. I feel competent in explaining the rationale of pharmacogenomics testing to patients.

| Strongly Disagree                                                                        Strongly Agree | | | | |
| --- | --- | --- | --- | --- |
| 1 | 2 | 3 | 4 | 5 |

1. I feel competent in discussing the risks and benefits of pharmacogenomics testing with patients.

| Strongly Disagree                                                                        Strongly Agree | | | | |
| --- | --- | --- | --- | --- |
| 1 | 2 | 3 | 4 | 5 |

**Section 3: Knowledge**

1. What may be the consequence of a pharmacogenomics polymorphism?

- An individual cannot metabolize any drugs
- An individual has a higher risk for toxicity when using prescription drugs
- A single drug dose is appropriate for a given indication
- Individualized dose adjustments should be made according to body surface area

1. What does a poor metabolizer phenotype indicate?

- Lower drug safety because of poor metabolism
- Good drug efficacy because of poor metabolism
- Decreased enzyme activity
- Increased enzyme activity

1. Which of the following is **not** correct about pre-emptive and reactive genotyping?

- Reactive genotyping is ordered as a drug therapy is being initiated or contemplated.
- Pre-emptive genotyping allows pharmacogenomics information to be available to guide prescribing.
- Reactive genotyping has been shown to be more cost-effective than pre-emptive genotyping.
- Pre-emptive genotyping usually tests for a panel of genes, whereas reactive genotyping usually tests for one to two genes.

1. Which of the following would be appropriate regarding clopidogrel therapy in patients who are CYP2C19 poor metabolizers?

- Initiate therapy with recommended starting dose
- Consider a 25% increase of recommended starting dose
- Consider a 25% decrease of recommended starting dose
- Consider alternative antiplatelet therapy if no contraindications

1. What sources have you used to learn about pharmacogenomics testing and its applications? (please select all that apply)

- Undergraduate education curriculum
- Postgraduate education curriculum
- Internet, website: __________________________
- Seminar, seminar name: __________________________
- Journal, journal name: __________________________
- Drug labels (package inserts)
- Colleague
- I have not learnt about pharmacogenomics testing and its applications
- Other (please specify): __________________________

**Section 4: Demographics**

1. Year of birth: _________

1. Gender: • Male • Female

1. Position:

- Doctor
- Pharmacist
- Nurse
- Other (please specify): __________________________

1. Main practice specialty: __________________________

1. Number of years of practice experience: __________________________

**Appendix 2: Post-training survey for TM1**

**Survey 2:** Post-training pharmacogenomics survey

Please rate the following (circle your answer):

**Section 1: Perceptions**

1. Pharmacogenomics is useful to my current practice.

| Strongly Disagree                                                                        Strongly Agree | | | | |
| --- | --- | --- | --- | --- |
| 1 | 2 | 3 | 4 | 5 |

1. I believe that a patient’s genetic profile may influence his/her response to drug therapy.

| Strongly Disagree                                                                        Strongly Agree | | | | |
| --- | --- | --- | --- | --- |
| 1 | 2 | 3 | 4 | 5 |

1. In general, the benefits of pharmacogenomics testing outweigh the risks.

| Strongly Disagree                                                                        Strongly Agree | | | | |
| --- | --- | --- | --- | --- |
| 1 | 2 | 3 | 4 | 5 |

1. Is pharmacogenomics testing useful for the following?

|  | Not useful at all                                 Extremely useful | | | | |
| --- | --- | --- | --- | --- | --- |
| (a) Identifying suitable medications for treatment | 1 | 2 | 3 | 4 | 5 |
| (b) Guide dosing of medications | 1 | 2 | 3 | 4 | 5 |
| (c) Reducing adverse drug reactions | 1 | 2 | 3 | 4 | 5 |
| (d) Improving treatment efficacy | 1 | 2 | 3 | 4 | 5 |
| (e) Reducing treatment costs | 1 | 2 | 3 | 4 | 5 |

**Section 2: Ability**

Please rank your perceived ability:

1. I feel competent in identifying clinical situations and/or patients in which pharmacogenomics testing is indicated.

| Strongly Disagree                                                                        Strongly Agree | | | | |
| --- | --- | --- | --- | --- |
| 1 | 2 | 3 | 4 | 5 |

1. I feel competent in interpreting results of pharmacogenomics tests.

| Strongly Disagree                                                                        Strongly Agree | | | | |
| --- | --- | --- | --- | --- |
| 1 | 2 | 3 | 4 | 5 |

1. I feel competent in making treatment recommendations based on results.

| Strongly Disagree                                                                        Strongly Agree | | | | |
| --- | --- | --- | --- | --- |
| 1 | 2 | 3 | 4 | 5 |

1. I can identify good pharmacogenomics resources (e.g. guidelines) for use clinically.

| Strongly Disagree                                                                        Strongly Agree | | | | |
| --- | --- | --- | --- | --- |
| 1 | 2 | 3 | 4 | 5 |

1. I feel competent in explaining the rationale of pharmacogenomics testing to patients.

| Strongly Disagree                                                                        Strongly Agree | | | | |
| --- | --- | --- | --- | --- |
| 1 | 2 | 3 | 4 | 5 |

1. I feel competent in discussing the risks and benefits of pharmacogenomics testing with patients.

| Strongly Disagree                                                                        Strongly Agree | | | | |
| --- | --- | --- | --- | --- |
| 1 | 2 | 3 | 4 | 5 |

**Section 3: Knowledge**

1. What may be the consequence of a pharmacogenomics polymorphism?

- An individual cannot metabolize any drugs
- An individual has a higher risk for toxicity when using prescription drugs
- A single drug dose is appropriate for a given indication
- Individualized dose adjustments should be made according to body surface area

1. What does a poor metabolizer phenotype indicate?

- Lower drug safety because of poor metabolism
- Good drug efficacy because of poor metabolism
- Decreased enzyme activity
- Increased enzyme activity

1. Which of the following is **not** correct about pre-emptive and reactive genotyping?

- Reactive genotyping is ordered as a drug therapy is being initiated or contemplated.
- Pre-emptive genotyping allows pharmacogenomics information to be available to guide prescribing.
- Reactive genotyping has been shown to be more cost-effective than pre-emptive genotyping.
- Pre-emptive genotyping usually tests for a panel of genes, whereas reactive genotyping usually tests for one to two genes.

1. Which of the following would be appropriate regarding clopidogrel therapy in patients who are CYP2C19 poor metabolizers?

- Initiate therapy with recommended starting dose
- Consider a 25% increase of recommended starting dose
- Consider a 25% decrease of recommended starting dose
- Consider alternative antiplatelet therapy if no contraindications

**Section 4: Needs assessment**

1. To better utilize pharmacogenomics information in the management of drug therapy, I would need… (please select all that apply)

- Better knowledge on pharmacology
- Better knowledge on drug metabolism
- Better knowledge on the basic concepts of pharmacogenomics
- Stronger evidence that pharmacogenomics improves clinical outcomes
- Better ability to apply my knowledge
- Better knowledge of legal regulations
- Support of my working institution
- Insurance coverage
- Expert counsel
- Other (please specify): __________________________

1. What is your preferred format for learning more about pharmacogenomics? (please select all that apply)

- Lectures
- Journal clubs
- Medical app
- E-learning
- Case discussion
- Other (please specify): __________________________

**Section 5: Evaluation of the training**

1. Please rate the following:

|  | Strongly disagree           Strongly agree | | | | |
| --- | --- | --- | --- | --- | --- |
| (a) The topics covered were relevant to me. | 1 | 2 | 3 | 4 | 5 |
| (b) The content was organized and easy to follow. | 1 | 2 | 3 | 4 | 5 |
| 1. The patient case aided my understanding of the clinical applications of pharmacogenomics. | 1 | 2 | 3 | 4 | 5 |
| 1. The training expanded my knowledge on pharmacogenomics. | 1 | 2 | 3 | 4 | 5 |

**Section 6: Demographics**

1. Within the past 6 months, how often have you ordered or recommended a pharmacogenomics test?

- 0
- 1 time per month
- 2-5 times per month
- >5 times per month

1. Do you anticipate ordering or recommending a pharmacogenomics test for a patient within the next 6 months?

- Yes
- No

1. Year of birth: _________

1. Gender: • Male • Female

1. Position:

- Doctor
- Pharmacist
- Nurse
- Other (please specify): __________________________

1. Main practice specialty: __________________________

1. Number of years of practice experience: __________________________

1. Please list any additional comments or feedback here:

|  |
| --- |

**Appendix 3: Pre-training survey for TM2**

**Nalagenetics Pharmacogenomics Pre-Training Survey**

We are Nalagenetics and we are creating a pharmacogenomics (PGx) program to integrate PGx into clinical practice. Pharmacogenomics is the study of how genes affect an individual’s response to drugs. One of the main benefits of PGx is to prevent and minimize adverse drug reactions (ADRs). This survey aims to gain feedback on our existing prototype PGx course. It will take approximately 10 minutes to complete, thank you for your time.

*Required

**Perceptions**

1.Are you currently integrating PGx into your clinical practice? *

a. Yes

b. No

2. Please rate the following: * (Mark only one oval per row.)

|  | Strongly disagree | Disagree | Neutral | Agree | Strongly agree |
| --- | --- | --- | --- | --- | --- |
| I am keen in adopting PGx into my clinical practice. |  |  |  |  |  |
| My patients experience ADRs from their prescribed medications. |  |  |  |  |  |
| I believe that a patient’s genetic profile may influence his/her response to drug therapy. |  |  |  |  |  |
| In general, the benefits of pharmacogenomics testing outweigh the risks. |  |  |  |  |  |
| I am keen in adopting PGx into my clinical practice. |  |  |  |  |  |
| My patients experience ADRs from their prescribed medications. |  |  |  |  |  |
| I believe that a patient’s genetic profile may influence his/her response to drug therapy. |  |  |  |  |  |
| In general, the benefits of pharmacogenomics testing outweigh the risks. |  |  |  |  |  |

3. PGx is useful for the following: * (Mark only one oval per row.)

|  | Strongly disagree | Disagree | Neutral | Agree | Strongly agree |
| --- | --- | --- | --- | --- | --- |
| (a) Choosing the best treatment option |  |  |  |  |  |
| (b) Optimizing drug dosing regimen |  |  |  |  |  |
| (c) Minimizing ADRs |  |  |  |  |  |
| (d) Improving treatment efficacy |  |  |  |  |  |
| (e) Reducing treatment costs |  |  |  |  |  |

4. Some of the obstacles involved in implementing PGx into my clinical practice are: * (Mark only one oval per row.)

|  | Strongly disagree | Disagree | Neutral | Agree | Strongly agree |
| --- | --- | --- | --- | --- | --- |
| (a) Cost |  |  |  |  |  |
| (b) Turn around time |  |  |  |  |  |
| (c) Lack of clear guidelines/evidence |  |  |  |  |  |
| (d) Confuse/worry patients |  |  |  |  |  |
| (e) PGx is not accurate |  |  |  |  |  |
| (f) PGx is not applicable to me |  |  |  |  |  |

**Ability**

5. If I were to adopt PGx into my clinical practice, I know how to do the following: * (Mark only one oval per row.)

|  | Strongly disagree | Disagree | Neutral | Agree | Strongly agree |
| --- | --- | --- | --- | --- | --- |
| (a) Identifying clinical situations/patients in which PGx is indicated |  |  |  |  |  |
| (b) Interpret PGx test results |  |  |  |  |  |
| (c) Make treatment recommendations based on PGx results |  |  |  |  |  |
| (d) Identify good PGx resources for clinical use |  |  |  |  |  |
| (e) Explain the rationale of PGx testing to my patients |  |  |  |  |  |
| (f) Discuss the risks and benefits of PGx testing |  |  |  |  |  |

**Knowledge**

6. What may be the consequence of a pharmacogenomics polymorphism? (Mark only one oval.)

1. An individual cannot metabolize any drugs
2. An individual has a higher risk for toxicity when using prescription drugs
3. A single drug dose is appropriate for a given indication
4. Individualized dose adjustments should be made according to body surface area

7. What does a poor metabolizer phenotype indicate? (Mark only one oval.)

1. Lower drug safety because of poor metabolism
2. Good drug efficacy because of poor metabolism
3. Decreased enzyme activity
4. Increased enzyme activity

8. Which of the following is not correct about pre-emptive and reactive genotyping? (Mark only one oval.)

1. Reactive genotyping is ordered as a drug therapy is being initiated or contemplated.
2. Pre-emptive genotyping allows pharmacogenomics information to be available to guide prescribing.
3. Reactive genotyping has been shown to be more cost-effective than pre-emptive genotyping.
4. Pre-emptive genotyping usually tests for a panel of genes, whereas reactive genotyping usually tests for one to two genes.

**Evaluation**

9.Please rate the following: * (Mark only one oval per row.)

|  | Strongly disagree | Disagree | Neutral | Agree | Strongly agree |
| --- | --- | --- | --- | --- | --- |
| The topics covered were relevant to me. |  |  |  |  |  |
| The content was organized and easy to follow. |  |  |  |  |  |
| The patient case aided my understanding of the clinical applications of pharmacogenomics. |  |  |  |  |  |
| The training expanded my knowledge on pharmacogenomics. |  |  |  |  |  |

**Appendix 4: Post-training survey for TM2**

**Nalagenetics Pharmacogenomics Post-Training Survey**

We are Nalagenetics and we are creating a pharmacogenomics (PGx) program to integrate PGx into clinical practice. Pharmacogenomics is the study of how genes affect an individual’s response to drugs. One of the main benefits of PGx is to prevent and minimize adverse drug reactions (ADRs). This survey aims to gain feedback on our existing prototype PGx course. It will take approximately 10 minutes to complete, thank you for your time.

*Required

1. Age Group: * Mark only one oval.

1. <30
2. 30-39
3. 40-49
4. 50-59
5. >60

2. Gender * Mark only one oval.

1. Female
2. Male
3. Prefer not to say
4. Other:

3. Number of practicing years * Mark only one oval.

1. <5
2. 5-10
3. 11-20
4. 21-30
5. >30

4. Where did you study medicine? * Mark only one oval.

1. Singapore
2. Overseas

5. What is your specialty?

6. I learnt about pharmacogenomics during my medical school * Mark only one oval.

1. Yes
2. No
3. I am not sure

**Perceptions**

7. Are you currently integrating PGx into your clinical practice? * Mark only one oval.

1. Yes
2. No

8. Please rate the following: * Mark only one oval per row.

|  | Strongly disagree | Disagree | Neutral | Agree | Strongly agree |
| --- | --- | --- | --- | --- | --- |
| I am keen in adopting PGx into my clinical practice. |  |  |  |  |  |
| My patients experience ADRs from their prescribed medications. |  |  |  |  |  |
| I believe that a patient’s genetic profile may influence his/her response to drug therapy. |  |  |  |  |  |
| In general, the benefits of pharmacogenomics testing outweigh the risks. |  |  |  |  |  |

9. PGx is useful for the following: * Mark only one oval per row.

|  | Strongly disagree | Disagree | Neutral | Agree | Strongly agree |
| --- | --- | --- | --- | --- | --- |
| (a) Choosing the best treatment option |  |  |  |  |  |
| (b) Optimizing drug dosing regimen |  |  |  |  |  |
| (c) Minimizing ADRs |  |  |  |  |  |
| (d) Improving treatment efficacy |  |  |  |  |  |
| (e) Reducing treatment costs |  |  |  |  |  |

10. Some of the obstacles involved in implementing PGx into my clinical practice are: * Mark only one oval per row.

|  | Strongly disagree | Disagree | Neutral | Agree | Strongly agree |
| --- | --- | --- | --- | --- | --- |
| (a) Cost |  |  |  |  |  |
| (b) Turn around time |  |  |  |  |  |
| (c) Lack of clear guidelines/evidence |  |  |  |  |  |
| (d) Confuse/worry patients |  |  |  |  |  |
| (e) PGx is not accurate |  |  |  |  |  |
| (f) PGx is not applicable to me |  |  |  |  |  |

**Ability**

11.If I were to adopt PGx into my clinical practice, I know how to do the following: * Mark only one oval per row.

|  | Strongly disagree | Disagree | Neutral | Agree | Strongly agree |
| --- | --- | --- | --- | --- | --- |
| (a) Identifying clinical situations/patients in which PGx is indicated |  |  |  |  |  |
| (b) Interpret PGx test results |  |  |  |  |  |
| (c) Make treatment recommendations based on PGx results |  |  |  |  |  |
| (d) Identify good PGx resources for clinical use |  |  |  |  |  |
| (e) Explain the rationale of PGx testing to my patients |  |  |  |  |  |
| (f) Discuss the risks and benefits of PGx testing |  |  |  |  |  |

**Knowledge**

12. What may be the consequence of a pharmacogenomics polymorphism? Mark only one oval.

1. An individual cannot metabolize any drugs
2. An individual has a higher risk for toxicity when using prescription drugs
3. A single drug dose is appropriate for a given indication
4. Individualized dose adjustments should be made according to body surface area

13. What does a poor metabolizer phenotype indicate? Mark only one oval.

1. Lower drug safety because of poor metabolism
2. Good drug efficacy because of poor metabolism
3. Decreased enzyme activity
4. Increased enzyme activity

14. Which of the following is not correct about pre-emptive and reactive genotyping? Mark only one oval.

1. Reactive genotyping is ordered as a drug therapy is being initiated or contemplated.
2. Pre-emptive genotyping allows pharmacogenomics information to be available to guide prescribing.
3. Reactive genotyping has been shown to be more cost-effective than pre-emptive genotyping.
4. Pre-emptive genotyping usually tests for a panel of genes, whereas reactive genotyping usually tests for one to two genes.

**Evaluation**

15.Please rate the following: * Mark only one oval per row.

|  | Strongly disagree | Disagree | Neutral | Agree | Strongly agree |
| --- | --- | --- | --- | --- | --- |
| The topics covered were relevant to me. |  |  |  |  |  |
| The content was organized and easy to follow. |  |  |  |  |  |
| The patient case aided my understanding of the clinical applications of pharmacogenomics. |  |  |  |  |  |
| The training expanded my knowledge on pharmacogenomics. |  |  |  |  |  |

**Appendix 5: TM2 Course content**

**Online course can be found here:** [**https://learning.nalagenetics.com/courses/course-v1:nalagenetics+PGX101+2020_Q1**](https://learning.nalagenetics.com/courses/course-v1:nalagenetics+PGX101+2020_Q1)

**Introduction**

Slides 1-3: Introduction of training, speaker and learning objectives

Slide 4: Current healthcare landscape

Slides 5-6: Introducing Nalagenetics

Slide 7: Content Outline

**Why do Pharmacogenomics Testing?**

Slide 8: Title slide

Slides 9-11: Strategies to reduce ADRs

Slide 10: Benefits of PGx testing

Slides 13-17: Reactive vs pre-emptive genotyping

Slides 18-22: Benefits of pre-emptive genotyping

Slides 23-26: Patient case scenario

**What Fundamentals of Pharmacogenomics I**

Slides 27-28: Title slide; Introduction of speaker

Slides 29-31: PGx background

Slides 32-38: PGx terms and definitions

Slides 39-41: Patient case scenario

**Where and How Fundamentals of Pharmacogenomics I: PGx resources**

Slide 42: Title slide

Slide 43: Overview

Slides 44-45: PharmGKB

Slides 46-48: CPIC

Slides 49-51: DPWG

Slide 52: Regulations

Slide 53: Scientific Evidence

Slides 54-57: Patient case scenario

**Who, When and How Important Pharmacogenes and How to Interpret Them**

Slide 58: Title slide

Slide 59: Overview

Slides 60-63: TCAs: amitriptyline and nortriptyline

Slides 64-67: Allopurinol and Steven Johnson Syndrome

Slides 68-73: Codeine

Slides 74-78: Clopidogrel

**Where and How How do I implement PGx into my Routine Clinical Practice?**

Slide 79: Title slide

Slides 80-83: Patient case scenario: conclusion

Slide 84: Nalagenetics workflow

Slides 85-90: Nalagenetics user interface

Slides 91-92: Conclusion
